# Guillain-Barré Syndrome Induced by Mycoplasma Pneumoniae: A Non-classical Presentation

**DOI:** 10.7759/cureus.83538

**Published:** 2025-05-05

**Authors:** Andrew M Hanchosky, Emily Hellmann, Christian Chong, Satish Sarvepalli

**Affiliations:** 1 Internal Medicine, Kettering Health, Dayton, USA; 2 Boonshoft School of Medicine, Wright State University, Dayton, USA; 3 Infectious Disease, Kettering Health, Dayton, USA

**Keywords:** acute inflammatory demyelinating polyneuropathy (aidp), acute motor axonal neuropathy (aman), csf composition, guillain-barre syndrome (gbs), mycoplasma pneumonia

## Abstract

This case report describes a 38-year-old Caucasian male patient with minimal past medical history who presented with worsening cough, shortness of breath, and a fever over a two-week time period. He then developed upper extremity weakness, lower extremity weakness, and respiratory failure. After he was stabilized in the intensive care unit, initial workup revealed positive immunoglobulin G antibodies to *Mycoplasma pneumonia *(*M. pneumonia*), and electromyography results indicated either acute motor axonal neuropathy or critical illness myopathy. Due to the patient’s clinical presentation and workup results, acute inflammatory demyelinating polyradiculoneuropathy was also included in the differential. After an extensive hospitalization, treatment with intravenous immunoglobulin, and repeat testing, we conclude that this was an atypical presentation of a Guillain-Barré syndrome variant secondary to *M. pneumoniae*.

## Introduction

Acute inflammatory demyelinating polyradiculoneuropathy (AIDP) is the most common subtype of Guillain-Barré syndrome (GBS), in which myelin sheaths and Schwann cells are injured due to an autoimmune-mediated inflammatory response that causes acute flaccid paralysis [1]. AIDP is typically secondary to a gastrointestinal or respiratory infection, with common culprits being *Campylobacter jejuni* (*C. jejuni*), cytomegalovirus, and *Mycoplasma pneumoniae* (*M. pneumoniae*) [1,2]. These infections initiate an immune response that targets peripheral nerves, leading to demyelination, slowed nerve conduction, and weakness. While the exact mechanism of this immune response is still being studied, recent research posits that the action of autoreactive T cells, due to cross-reactivity between infectious sources and peripheral nerve elements, may be the primary offender [1].

AIDP classically presents with ascending symmetric muscle weakness starting in the lower extremities and progressing proximally. It can affect the respiratory muscles, causing respiratory failure in about 25% of patients [3]. Cranial nerves can also be affected, with one study showing that 47% of patients with AIDP developed cranial nerve palsies [4]. However, the presentation can be variable, complicating the diagnosis and treatment. Cases have been reported with diverse initial manifestations including abdominal pain, descending weakness, back pain, facial weakness, and hyperreflexia [5-7].

Acute motor axonal neuropathy (AMAN) is a pure motor axonal variant of GBS characterized by acutely progressive weakness, ataxia, areflexia, and a lack of sensory symptoms [8]. In contrast with other forms of GBS, AMAN is a noninflammatory disorder that can produce selective degradation of the axons of motor neuron roots and nerves without demyelination [9]. Additionally, AMAN progresses more rapidly, has an earlier peak of symptoms compared to demyelinating variants of GBS, spares deep tendon reflexes, and rarely exhibits autonomic dysfunction [8]. Immunoglobulin (Ig) G binds to GM1 at the axolemma and activates the complement system, ultimately leading to nerve conduction failure by interfering with sodium channels and the axoglial junction [9]. *C. jejuni* is known to be one of the main causes, with the use of molecular mimicry as the bacteria’s lipo-oligosaccharides mimic humans’ gangliosides [8].

We present a case of GBS triggered by *M. pneumoniae* with a non-classic manifestation. Instead of the typical symmetric ascending weakness, our patient initially experienced right-sided sensory deficits and bilateral upper extremity weakness. This case highlights the importance of considering less common presentations of GBS, as early treatment with intravenous IgG or plasma exchange therapy is critical in these patients [1].

## Case presentation

A 38-year-old Caucasian male with a history of tobacco use disorder (one pack per day for unknown number of years), prior alcohol use disorder (10 year duration, quit one year ago), and class one obesity presented to the emergency department (ED) with a chief complaint of worsening cough and shortness of breath for approximately one week. Four days prior, he visited an urgent care and was prescribed azithromycin, Tessalon Perles, and an albuterol inhaler for suspected otitis media and acute bronchitis. This did not alleviate his symptoms. In the ED, he described new symptoms of right-hand weakness, numbness in his right toe, and right shoulder pain. He was diagnosed with radiculopathy for which he was given a corticosteroid taper and pain medication. Due to worsening symptoms, he returned to the emergency department that evening with a new chief complaint of worsening generalized weakness. He was diagnosed with a viral syndrome. As he was about to be discharged, he had a syncopal episode along with emesis and urinary incontinence. He then started to appear cyanotic with an oxygen saturation of around 40%. He was placed on supplemental oxygen via facemask, which was later switched to bilevel positive airway pressure (BiPAP).

**Figure 1:**
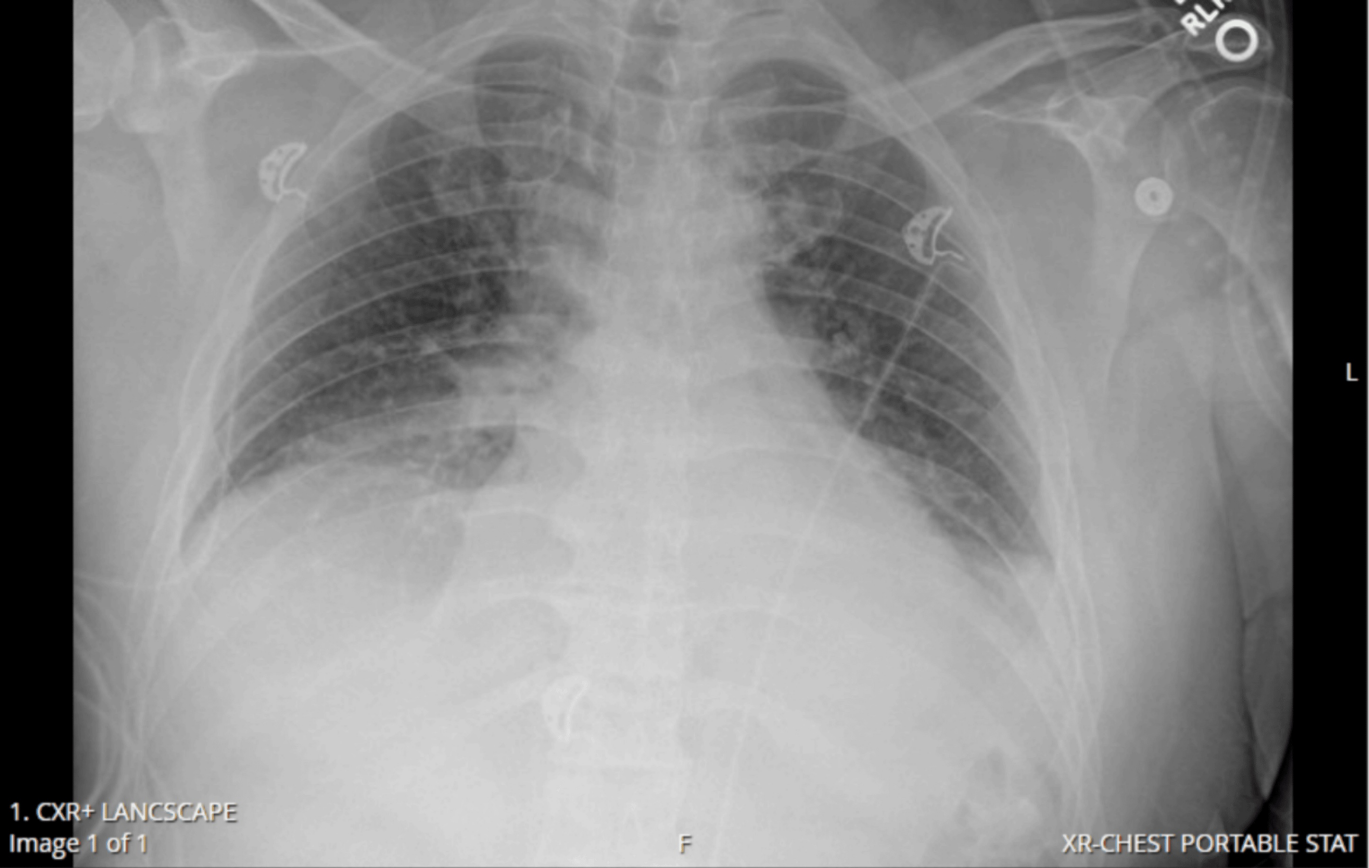
Chest x-ray just prior to intubation, revealing small bilateral pleural effusions with bibasilar airspace disease.

Initial imaging in the emergency department included computed tomography (CT) of the head, which revealed no abnormalities, and a CT pulmonary angiogram revealed bibasilar pneumonia. An ABG revealed a mixed respiratory/metabolic acidosis with an elevated lactate of 4.2 mmol/L. He also had a mild leukocytosis of 12.3 k/µL. Due to worsening oxygen saturation, he was transferred to a higher-level intensive care unit (ICU). Upon arrival in the ICU, the patient then developed bilateral upper extremity pain and weakness. Physical exam revealed bilateral upper extremities with gross sensation intact, however, there was no withdrawal from painful stimuli and no resistance to gravity. Strength and sensation were intact in the bilateral lower extremities. An arterial blood gas was drawn while on BiPAP, which revealed persistent hypercapnia. Over the next eight hours, he developed bilateral lower extremity weakness with worsening hypoxia and tachypnea. The severity of his respiratory distress did not seem to correlate with his chest x-ray (Figure 1). Repeat physical exam revealed flaccid paralysis of the left upper and left lower extremities with absent bilateral patellar and Achilles deep tendon reflexes. He agreed to intubation due to his worsening respiratory status. He was started on ceftriaxone, azithromycin, vancomycin, acyclovir, and methylprednisolone to cover for community-acquired pneumonia and suspected meningitis.

Over the next day, he required maximum ventilator settings along with epoprostenol to maintain adequate oxygenation. Infectious disease and neurology were consulted. Additional imaging studies included magnetic resonance imaging of the head and cervical spine, which revealed no acute abnormalities. An electroencephalogram was performed, which showed no sign of seizure activity. Initial laboratory workup was positive for *M. pneumoniae* via upper respiratory polymerase chain reaction (PCR). Follow-up with* M. pneumoniae* antibodies revealed positive for IgG yet negative for IgM. Blood cultures, bronchoalveolar lavage, and sputum cultures were negative for pathogenic organisms. Results of the lumbar puncture on Day 2 of hospitalization (Table 1) did not indicate GBS or meningitis. Cerebrospinal fluid (CSF) and blood cultures were all negative, including *M. pneumoniae* PCR of CSF. Other negative test results included ganglionic acetylcholine receptor antibodies, myositis panel, GM1 IgG autoantibody, human immunodeficiency virus, and hepatitis panel. During the first couple of days, he had no major changes in his physical exam findings.

**Table 1:** CSF results on hospital Day 1 and Day 17. * Results from two different CSF fluid collection tubes. CSF: cerebrospinal fluid

CSF Analysis	Day 1	Day 17
Glucose (40 - 70 mg/dL)	113 mg/dL	94 mg/dL
Protein (15 - 45 mg/dL)	26 mg/dL	109 mg/dL
Appearance	Clear	Clear
Color	Colorless	Colorless
Total Nucleated Cells (< 5 cells/µL)	0 cells/µL	9, 7 cells/µL*
Lymphocytes (< 5 cells/µL)	0 cells/µL	70, 68 cells/µL*
Monocytes (< 5 cells/µL)	0 cells/µL	30, 32 cells/µL*
Red Blood Cells (0 - 10 cells/µL)	7, 5 cells/µL*	0 cells/µL

Initial electromyography (EMG) and nerve conduction study (NCS) were completed on hospital Day 4. Ulnar nerve sensory conduction was found to be normal in amplitude and reproducibility, with motor amplitudes decreased on the left and borderline low on the right. Bilateral tibial nerve motor conduction had decreased amplitudes as well. Bilateral sural nerve sensory conduction revealed normal findings. Radial and median nerve conduction could not be performed due to electrical interference. Overall, the studies showed a mild-to-moderate decrease in nerve conduction amplitudes without noticeable denervation potentials, indicating critical illness myopathy versus AMAN. He was started on intravenous immunoglobulin for a three-day course, however, no significant improvement was seen on the physical exam during those three days. Repeat EMG and NCS on Day 11 revealed similar findings to those of prior tests. Repeat lumbar puncture was performed on Day 17, with results also shown in Table 1.

Even though the patient’s ventilator requirements decreased during his stay, he continued to require ventilator support. In order to participate in physical and occupational therapy, he agreed to a tracheostomy. When he was transferred out of the ICU on Day 20, he was able to tolerate noninvasive ventilation. He slowly started to regain function in his upper extremities, including increased hand grip strength and increased upper extremity range of motion. However, improvement in his lower extremities was initially delayed. By Day 20, he was able to lift all extremities against gravity. He was then transferred to a long-term care facility.

## Discussion

Our patient displayed bilateral upper extremity weakness before the onset of lower extremity weakness when he was admitted to the ICU. The initial physical examination conducted in the ED before transfer did not note any strength or sensation deficits in the bilateral upper or lower extremities. This type of presentation is atypical for AIDP and is more commonly documented in cases of Miller-Fisher Syndrome. However, these patients have at least two out of three findings of areflexia, ataxia, or ophthalmoplegia, which did not accurately fit our patient’s presentation [10].

Our patient’s weakness was reported as generalized without any focal findings of decreased strength or sensation. This was likely due to the timing of his presentation, as he decompensated rapidly after his initial ED visit. This emphasizes the utility and importance of a thorough physical examination with every patient encounter. If our patient had exhibited signs of gross extremity weakness or changes in sensation, admission and proper treatment could have been administered more promptly. As our patient decompensated, he developed a descending weakness of his right side more so than his left. The common presentation of patients with GBS includes ascending weakness leading to flaccid paralysis and hyporeflexia or areflexia in one to six weeks after a potential trigger [11]. This highlights the various ways that GBS subtypes can present, as the typical ascending weakness may not always be seen. He also presented with concurrent severe respiratory distress, which rapidly led to respiratory failure. This has been noted to occur in approximately 25% of patients with GBS [12].

As in our patient, where the initial diagnosis is largely unclear, the Brighton criteria can be utilized to assist in the diagnosis of GBS, which includes four different levels of diagnostic certainty. The Brighton criteria include: bilateral flaccid limbs, decreased or absent deep tendon reflexes, no other alternative diagnosis, monophasic timeframe of 12 hours to 28 days from initial symptom onset to severe symptoms, NCS findings consistent with GBS, elevated CSF protein, and CSF cell count <50 µL [13]. According to the Brighton criteria, our findings indicate a diagnostic certainty level of two with results of both lumbar punctures [13].

Our patient was among the approximately 10% of GBS patients who have a normal initial CSF analysis rather than classic albuminocytologic dissociation [10]. However, his overall clinical picture strongly pointed to a neurological pathology. The lack of definitive CSF findings placed greater emphasis on the EMG and NCS results. Although the initial EMG in this case was not enough to confirm the diagnosis, as is commonly seen with AIDP [14]. As indicated by Leonhard et al., an initial EMG/NCS with normal results is not enough to rule out GBS [15]. The initial and subsequent EMG/NCS findings in our patient revealed a mixed picture and did not definitively suggest a singular diagnosis. The second lumbar puncture did show albuminocytologic dissociation, which helped confirm the diagnosis of GBS.

Infection is the most common etiology of GBS, with 75% of AIDP cases involving an infectious prodrome [16]. This was seen in our patient with his diagnosis of acute bronchitis. Among infectious organisms,* C. jejuni *is the most common, as it causes 30% of reported cases, with *M. pneumoniae* being the second most common organism [17].* M. pneumoniae* is a bacterium known to elicit a potential autoimmune response. These autoantibodies and inflammatory responses can result in damage to multiple organ systems, including the integumentary, cardiac, gastrointestinal, and musculoskeletal which can manifest as muscle weakness [18].

The GBS subtype to which our patient aligns presents with a mixed picture. The most common subtype of GBS is AIDP in the United States. However, the EMG/NCS results did not reveal a demyelinating process, pointing more towards AMAN. Our patient had decreased patellar and Achilles tendon reflexes, which is not expected with AMAN. However, as AMAN purely affects motor neurons, our patient’s lack of withdrawal to pain strongly suggests against AMAN. Our patient also lacked positive GM1 IgG autoantibodies, which can be seen in 50% of cases of acute motor neuropathy [19]. A diagnosis of acute motor and sensory axonal neuropathy would better match the EMG/NCS results along with our patient’s presentation. In situations like this, where there are conflicting results, another EMG/NCS in the future would likely help elucidate the diagnosis.

## Conclusions

This case highlights the complexities involved in diagnosing GBS, especially when presenting with atypical features. Our patient’s progression from generalized weakness to bilateral upper and lower extremity paralysis, coupled with severe respiratory distress, underscores the variable nature of GBS subtypes. The overlapping clinical signs, particularly in cases triggered by infections like *M. pneumoniae*, can lead to diagnostic uncertainty. This case also emphasizes the importance of timely and repeated diagnostic testing, including CSF analysis and EMG, to accurately identify the GBS variant.

Given the autoimmune potential of *M. pneumoniae*, it is critical to consider it as a trigger for GBS, even when the clinical presentation deviates from the typical ascending weakness pattern. Early recognition and prompt intervention with therapies such as intravenous immunoglobulin or plasma exchange are crucial in improving patient outcomes. As GBS can manifest in various forms, healthcare providers must maintain a high index of suspicion and be prepared to adapt treatment based on evolving symptoms and diagnostic findings.
